# Molecular Prevalence, Genotypic Distribution and Zoonotic Potential of *Enterocytozoon bieneusi* in Hu Sheep and Goats in Southern Zhejiang Province, Eastern China

**DOI:** 10.3390/vetsci13090847

**Published:** 2026-08-22

**Authors:** Yongli Jian, Houqiang Luo, Qingsong Han, Zhongkai Zhang, Longchuan Duan, Luying Yang, Meng Wang, Peide Li, Xingyang Cui, Yongan Gao, Haiyang Song, Yiqiang Tu, Suzhen Liu, Wei Zhao

**Affiliations:** 1 College of Animal Science, Wenzhou Vocational College of Science and Technology, Wenzhou 325006, China; wkyjianyl@163.com (Y.J.); chviolet1984@sina.com (H.L.); hanqingsong@wzvcst.edu.cn (Q.H.); duanlongchuan@wzvcst.edu.cn (L.D.); mengwang11272022@163.com (M.W.); su-2737@163.com (P.L.); cxy_2010@126.com (X.C.); gaoyongan79@163.com (Y.G.); haiyangsong125@163.com (H.S.); 2 The First Clinical Medical College, Wenzhou Medical University, Wenzhou 325035, China; 15268405497@163.com; 3 Animal Disease Prevention and Control Center of Xianju County, Taizhou 317300, China; 13967619890@163.com

**Keywords:** *Enterocytozoon bieneusi*, Hu sheep, goat, ITS genotype, zoonosis, southern Zhejiang

## Abstract

*Enterocytozoon bieneusi* is a major zoonotic pathogen causing human microsporidiosis, with small ruminants serving as crucial reservoir hosts. Hu sheep and local goats are pillar livestock breeds in Zhejiang Province, yet no parallel comparative epidemiological data on *E. bieneusi* exist for sympatric small ruminants in southern Zhejiang, and the infection status in Hu sheep remains entirely uncharacterized. Here, a total of 297 fecal samples (159 Hu sheep, 138 goats) were collected from four administrative regions in southern Zhejiang from 2021 to 2023 and screened via nested PCR targeting the ribosomal internal transcribed spacer (ITS) region of the *E. bieneusi* genome. Overall, the total *E. bieneusi* prevalence reached 18.18%, and Hu sheep exhibited a significantly higher infection rate (24.53%, 39/159) than sympatric goats (10.87%, 15/138; χ^2^ = 9.27, *p* = 0.002). Obvious geographical and age-dependent disparities were observed in Hu sheep, with pre-weaning lambs (<3 months) showing the highest prevalence (60.42%), while adult sheep (>1 year) had the lowest rate (4.48%). No significant age or regional differences were detected in goats. Five genotypes were identified from Hu sheep: CM7 (*n* = 25), BEB6 (*n* = 10), CHG1 (*n* = 2), Type IV (*n* = 1), and CHG5 (*n* = 1). Five genotypes were detected from goats: CHG1 (*n* = 9), BEB6 (*n* = 3), Type IV (*n* = 1), CHG3 (*n* = 1), and CYG-2 (*n* = 1). BEB6, Type IV, and CHG1 were shared by both hosts. All genotypes belonged to zoonotic Group 1 and Group 2. This study provides the first systematic parallel survey of *E. bieneusi* in sympatric Hu sheep and goats in eastern China. It identifies distinct host-specific infection patterns potentially associated with divergent rearing systems, provides molecular data consistent with potential inter-species circulation and shared environmental exposure, and highlights the need for breed-tailored biosecurity strategies within a One Health framework.

## Simple Summary

*Enterocytozoon bieneusi* is a widespread zoonotic pathogen that causes intestinal disease in humans and various animal species, representing a notable concern for both public health and livestock farming. Small ruminants such as sheep and goats are well-documented reservoir hosts for this pathogen. In southern Zhejiang Province, eastern China, Hu sheep and local goats are major livestock breeds raised under different rearing systems, yet systematic baseline data on *E. bieneusi* infection in these populations are still lacking. In this work, 297 fecal samples were collected from seven commercial farms across southern Zhejiang from 2021 to 2023, and analyzed via molecular detection and genotyping methods. The overall prevalence of *E. bieneusi* was 18.18%, with a significantly higher infection rate in Hu sheep than in goats. Pre-weaning lambs showed the highest infection susceptibility among Hu sheep. All seven identified genotypes have zoonotic potential, and three genotypes were shared by both host species, suggesting possible shared environmental exposure or inter-species circulation. These findings provide scientific support for developing breed-specific on-farm biosecurity strategies and mitigating zoonotic public health risks in the region.

## 1. Introduction

Microsporidia constitute a diverse group of spore-forming obligate intracellular fungi capable of infecting a wide range of invertebrate and vertebrate hosts. Of the at least 17 microsporidian species known to cause human disease, *Enterocytozoon bieneusi* is responsible for over 90% of clinical microsporidiosis cases globally [1,2]. In immunocompetent individuals, infection with *E. bieneusi* typically causes self-limiting diarrhea or remains subclinical. In contrast, immunocompromised groups, including people living with HIV/AIDS, solid organ transplant recipients and malnourished children, are susceptible to severe, persistent complications such as life-threatening chronic diarrhea and wasting syndrome, accompanied by multi-organ disseminated infection [3,4]. The environmentally resilient spores of *E. bieneusi* transmit primarily via the fecal–oral route, through ingestion of contaminated water or food, or direct contact with infected humans and animals [5]. Multiple waterborne and foodborne outbreaks caused by *E. bieneusi* have been documented across the globe, highlighting its severe risks to food and drinking water safety [6,7]. Accordingly, *E. bieneusi* has been designated a Category B priority pathogen by the U.S. National Institute of Allergy and Infectious Diseases (NIAID), while the U.S. Environmental Protection Agency (EPA) lists it as a high-priority waterborne microbial contaminant, underscoring the necessity of sustained surveillance and targeted intervention strategies for microsporidiosis in public health programs [2].

The internal transcribed spacer (ITS) region of the small subunit ribosomal RNA (*SSU rRNA*) gene serves as the universally recognized gold-standard molecular marker for *E. bieneusi* genotyping and molecular epidemiological tracking [8]. To date, more than 860 distinct ITS genotypes of *E. bieneusi* have been recovered from over 210 animal species across 50 countries, which can be phylogenetically partitioned into 15 well-supported monophyletic groups [2]. Group 1 encompasses the majority of confirmed zoonotic variants, including D, Type IV, EbpA and EbpC, all of which circulate widely among humans and diverse domestic and wild animal hosts [3]. Group 2 was long considered ruminant-specific; however, accumulating clinical evidence from human microsporidiosis patients has verified that genotypes BEB6, I and J also possess zoonotic transmissibility [3]. In contrast, genotypes assigned to Groups 3–15 generally exhibit pronounced host restriction and pose minimal threats to human populations [2,3]. Such inter-group divergence in host tropism creates unequal public health burdens across different animal reservoirs. Accordingly, livestock species with frequent human contact and high fecal spore shedding loads should be prioritized in routine pathogen surveillance frameworks.

Domestic sheep and goats constitute critical natural reservoirs of *E. bieneusi* worldwide. A global systematic meta-analysis reported pooled molecular prevalence estimates of 17.4% and 16.3% for sheep and goats, respectively [9]. To date, a total of 69 ITS genotypes have been characterized from sheep, alongside 41 distinct genotypes recovered from goat populations. Notably, nearly all these ruminant-derived genotypes cluster within zoonotic Group 1 or Group 2 clades, underscoring the vital epidemiological role of small ruminants as sources of cross-species spillover to human communities and inter-animal pathogen transmission [2,9]. China sustains one of the world’s largest small ruminant populations, and intensive sheep and goat production constitutes a vital pillar of rural livelihoods across numerous provincial regions. Over the past decade, nationwide molecular epidemiological investigations of ovine and caprine *E. bieneusi* have been carried out in more than 20 provinces [10]. Distinct regional genotypic disparities have been identified: CM7 and BEB6 dominate sheep herds across northern and western China, while CHG1 and CHG3 represent the most prevalent variants in goat populations from central and southern provinces [10,11]. The consistent detection of zoonotic *E. bieneusi* genotypes in Chinese sheep and goats has aroused persistent public health concerns regarding occupational exposure among farmers and veterinary practitioners, as well as environmental spore contamination that threatens adjacent residential populations.

Situated on the southeastern coast of China, Zhejiang Province features a subtropical monsoon climate with high ambient temperatures and abundant annual precipitation. Such climatic conditions may prolong the environmental survival of *E. bieneusi* spores and could potentially increase pathogen transmission risk [10]. Small ruminant production constitutes a vital component of Zhejiang’s livestock economy. Hu sheep, an indigenous Chinese breed prized for high-quality lamb pelts and tender meat, are reared across the province under intensive fully enclosed housing systems [12]. In contrast, goat production is concentrated in the hilly uplands of southern Zhejiang, where small-scale semi-free-range husbandry is the predominant practice. Despite the prominent economic value of local ovine and caprine industries, systematic epidemiological baseline data concerning *E. bieneusi* circulation in this region remain largely absent [10]. To date, only one study in Zhejiang Province has investigated *E. bieneusi* in intensively farmed goats [10]; no systematic epidemiological work has focused on Hu sheep or cohabiting free-range goat herds across southern Zhejiang. This lack of regional baseline data hinders evidence-based disease intervention and quantitative assessment of local zoonotic public health risks.

Unlike previous single-host surveys, the present study adopted a parallel comparative design targeting sympatric Hu sheep (intensive indoor housing) and local goats (semi-free-range husbandry) in southern Zhejiang, with four specific objectives: (i) to determine and compare the molecular prevalence of *E. bieneusi* between the two small ruminant species; (ii) to characterize genotypic profiles and host-specific dominance patterns of circulating isolates; (iii) to assess zoonotic risk and potential inter-species circulation via phylogenetic analysis and shared genotype identification; and (iv) to dissect epidemiological drivers associated with rearing systems and host demographics, so as to provide evidence-based guidance for site-specific pathogen control.

## 2. Materials and Methods

### 2.1. Ethics Approval

The sampling protocol of this study was reviewed and approved by the Research Ethics Committee of Wenzhou Medical University (Approval No. SCILLSC-2021-01; approval date: 3 April 2020). Oral consent was obtained from all farm operators prior to fecal sample collection. This consent procedure was approved by the institutional review board, as the study involved only non-invasive fecal sampling with no human or animal research interventions. Verbal consent was documented in field sampling records, and was used because many participating farmers had limited literacy and preferred verbal confirmation over formal written documentation. No invasive animal manipulations were conducted throughout the study.

### 2.2. Study Area and Sample Collection

**Figure 1:**
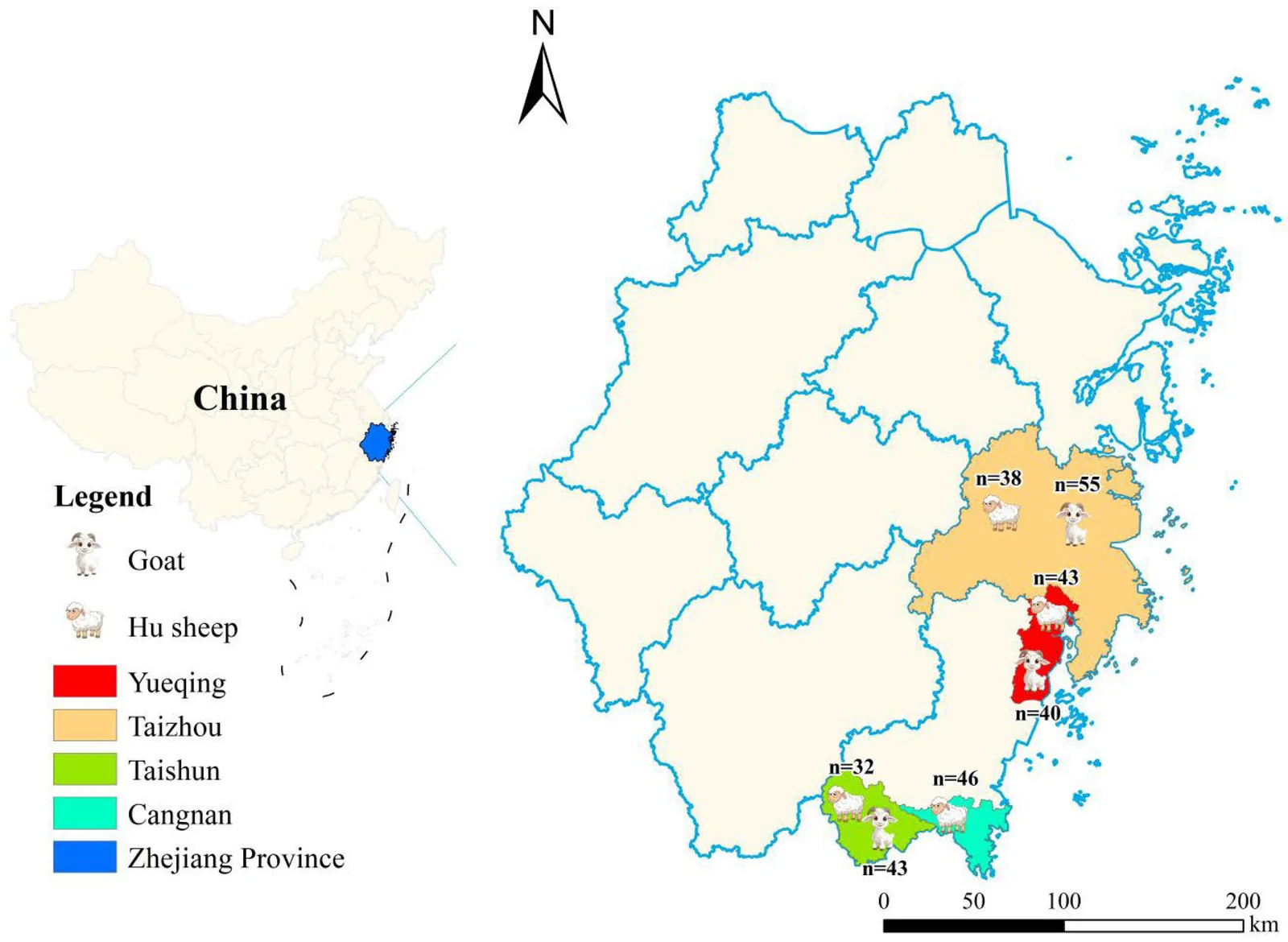
Sampling distribution across southern Zhejiang. Paired values indicate the number of fecal samples collected from Hu sheep and goats in each investigated region.

Between September 2021 and May 2023, 297 fresh fecal specimens were randomly collected from clinically healthy animals at seven commercial farms. Specifically, four Hu sheep farms (one per sampled county) and three goat farms (one per sampled county) were enrolled. All Hu sheep farms operated intensive fully housed production systems with herd sizes of 200–500 breeding ewes; all goat farms adopted semi-free-range management with flock sizes of 80–200 adult goats. These included 159 Hu sheep samples from four counties and county-level cities in southern Zhejiang: 32 in Taishun County, 46 in Cangnan County, 43 in Yueqing City, and 38 in Taizhou City. Additionally, 138 goat samples were collected from three regions: 43 in Taishun County, 40 in Yueqing City, and 55 in Taizhou City; no goat farms were sampled in Cangnan due to logistical limitations, as no eligible commercial goat farms in the county agreed to participate during the study period (Figure 1). Hu sheep were stratified into three age cohorts: pre-weaning lambs (<3 months, *n* = 48), post-weaning fattening sheep (4 months to 1 year, *n* = 44), and adult breeding ewes (>1 year, *n* = 67). This fine stratification was possible because participating Hu sheep farms maintained complete individual birth and breeding records. Due to incomplete breeding records, goats were divided into two broad groups: juveniles (<1 year, *n* = 62) and mature adults (>1 year, *n* = 76). This mismatch in age stratification limits direct interspecies comparison of age-related infection patterns.

Approximately 10–30 g of fresh feces was collected immediately post-defecation using sterile disposable latex gloves and transferred to individually labeled sterile centrifuge tubes. Specimens were transported on ice and delivered to the laboratory within 48 h, then stored at 4 °C prior to DNA extraction.

### 2.3. Genomic DNA Extraction

Fecal samples were homogenized and sieved through a 45 μm mesh filter to remove coarse plant fiber debris. The filtrate was concentrated via centrifugation at 1500× *g* for 10 min. Genomic DNA was extracted from 200 mg concentrated fecal sediment using the QIAamp DNA Stool Mini Kit (Qiagen, Hilden, Germany) following the manufacturer’s standard protocol, with an elevated lysis temperature of 95 °C implemented to maximize spore lysis and DNA recovery. Purified DNA was quantified and preserved at −20 °C for downstream molecular assays.

### 2.4. Nested PCR Amplification

Nested PCR targeting the ITS region of the *SSU rRNA* gene was used for molecular detection of *E. bieneusi*, with primer sequences, reaction systems, and thermal cycling protocols strictly implemented according to the previously validated classic method [13]. The first-round amplification adopted outer primers EBITS3 (5′-GGTCATAGGGATGAAGAG-3′) and EBITS4 (5′-TTCGAGTTCTTTCGCGCTC-3′), while the second-round nested amplification used inner primers EBITS1 (5′-GCTCTGAATATCTATGGCT-3′) and EBITS2.4 (5′-ATCGCCGACGGATCCAAGTG-3′), yielding a target fragment of approximately 390 bp. Each 25 μL PCR reaction system contained 1× Ex Taq buffer (supplemented with Mg^2+^), 0.2 mM dNTP mixture, 0.4 μM of each primer, 1.25 U TaKaRa Ex Taq DNA polymerase (TaKaRa Bio Inc., Tokyo, Japan), and 2 μL template DNA. The first-round thermal procedure consisted of an initial denaturation at 95 °C for 5 min, followed by 35 cycles of 94 °C for 30 s, 57 °C for 30 s, and 72 °C for 40 s, with a final extension at 72 °C for 10 min. The second-round PCR maintained identical reaction components and cycling parameters, except for a reduced annealing temperature of 55 °C. Rigorous quality control was performed for each PCR batch, with sterile distilled water as the negative control and genomic DNA of the *E. bieneusi* Peru11 genotype as the positive control. All samples were amplified in duplicate to prevent false-negative results. Amplified products were visualized by 1.5% agarose gel electrophoresis with GelRed staining (Biotium Inc., Hayward, CA, USA) under UV illumination.

### 2.5. Sanger Sequencing and Genotype Assignment

All positive secondary PCR products were purified and subjected to bidirectional Sanger sequencing by Sangon Biotech (Shanghai, China). Raw sequencing chromatograms were manually inspected and trimmed using ChromasPro software v2.2.0 (Technelysium Pty Ltd., Brisbane, Australia; https://technelysium.com.au/wp/chromaspro/, accessed on 20 July 2026), and consensus sequences were assembled with DNASTAR Lasergene v7.1.0 (DNASTAR Inc., Madison, WI, USA; https://www.dnastar.com/, accessed on 20 July 2026). Genotypes were identified via BLAST+ 2.17.0 homology searches against validated reference ITS sequences deposited in the NCBI GenBank database (https://blast.ncbi.nlm.nih.gov/Blast.cgi, accessed on 20 July 2026). Consistent with universal *E. bieneusi* nomenclature guidelines, only the 243 bp core ITS segment was used for genotyping; sequences sharing 100% nucleotide identity with published reference strains were assigned corresponding formal genotype designations. Representative sequences of all identified genotypes were deposited in GenBank. For genotypes shared by Hu sheep and goats (BEB6, CHG1, Type IV), one representative sequence from each host species was submitted independently to document cross-host circulation. The accession numbers are PZ670828–PZ670837.

### 2.6. Phylogenetic Analysis

Reference ITS sequences representing major *E. bieneusi* phylogenetic groups were retrieved from GenBank. Multiple sequence alignment was conducted using Clustal X v2.1. A neighbor-joining (NJ) phylogenetic tree was constructed in MEGA 7.0 software employing the Kimura 2-parameter substitution model. Bootstrap analysis with 1000 replicates was performed to evaluate the robustness of tree topologies. All detected genotypes were assigned to phylogenetic groups based on their clustering patterns with established reference isolates.

### 2.7. Statistical Analyses

*E. bieneusi* prevalence was calculated as the proportion of PCR-positive samples relative to the total number of tested specimens, expressed as percentages, with 95% confidence intervals (95% CIs) calculated using the Wilson score method. Chi-square (χ^2^) tests were performed in SPSS version 22.0 (SPSS Inc., Chicago, IL, USA) to compare infection rates across geographical regions and age cohorts. Fisher’s exact test was applied instead when the expected frequency in any cell was less than 5. A two-tailed *p*-value < 0.05 was defined as statistically significant, and *p* < 0.01 was regarded as highly significant.

## 3. Results

### 3.1. Overall Prevalence of E. bieneusi

In total, 54 out of 297 fecal samples tested positive for *E. bieneusi* via nested PCR, yielding an overall regional prevalence of 18.18% (95% CI: 14.0–23.2%, 54/297) (Table 1). All statistical comparisons presented herein are descriptive and observational, and do not establish causal relationships. The infection rate was significantly higher in Hu sheep (24.53%, 95% CI: 18.2–32.0%, 39/159) than in sympatric goats (10.87%, 95% CI: 6.6–17.3%, 15/138), with a statistically significant inter-species difference (χ^2^ = 9.27, df = 1, *p* = 0.002).

Highly significant inter-regional disparities in *E. bieneusi* prevalence were detected among Hu sheep (χ^2^ = 26.41, df = 3, *p* < 0.001). Cangnan County recorded the highest infection rate at 43.48% (95% CI: 29.8–58.0%, 20/46), followed by Taishun County (40.63%, 95% CI: 25.5–57.8%, 13/32), Yueqing City (9.30%, 95% CI: 3.7–21.9%, 4/43), and Taizhou City (5.26%, 95% CI: 1.5–17.3%, 2/38) (Table 1). For goats, prevalence fluctuated across sampling sites from 7.50% (Yueqing, 3/40, 95% CI: 2.6–20.0%) to 14.55% (Taizhou, 8/55, 95% CI: 7.3–27.0%) (Table 1), with no statistically significant differences observed across regions (χ^2^ = 1.34, df = 2, *p* = 0.510).

Highly significant age-dependent differences were observed in Hu sheep (χ^2^ = 49.71, df = 2, *p* < 0.001). Pre-weaning lambs aged < 3 months exhibited the highest infection rate of 60.42% (95% CI: 45.6–73.6%, 29/48), followed by post-weaning sheep aged 4 months to 1 year (15.91%, 95% CI: 7.9–29.6%, 7/44), while adult sheep > 1 year old showed the lowest susceptibility (4.48%, 95% CI: 1.4–12.5%, 3/67) (Table 1). In goats, infection rates were comparable between juveniles aged < 1 year (11.29%, 95% CI: 5.4–22.2%, 7/62) and mature adults > 1 year old (10.53%, 95% CI: 5.2–20.1%, 8/76) (Table 1), with no statistically significant age-related divergence (χ^2^ = 0.02, df = 1, *p* = 0.888).

### 3.2. Genotypic Distribution of E. bieneusi

Complete Sanger sequencing was successfully accomplished for all 54 PCR-positive samples, identifying seven distinct known ITS genotypes in total. Five genotypes were recovered from Hu sheep: CM7 (*n* = 25), BEB6 (*n* = 10), CHG1 (*n* = 2), Type IV (*n* = 1), and CHG5 (*n* = 1). CM7 constituted the dominant genotype, accounting for 64.1% (25/39) of all ovine positive isolates, followed by BEB6 (25.6%, 10/39). Three rare genotypes (CHG1, Type IV, CHG5) were sporadically detected, each represented by only one or two isolates. CM7 and BEB6 were widely distributed across all surveyed regions and age groups, whereas minor genotypes occurred as scattered single isolates (Table 1). Five genotypes were identified in goats: CHG1 (n = 9), BEB6 (*n* = 3), Type IV (*n* = 1), CHG3 (*n* = 1), and CYG-2 (*n* = 1). Genotype CHG1 dominated caprine positive samples at a frequency of 60.0% (9/15), followed by genotype BEB6 (20.0%, 3/15). Genotypes Type IV, CHG3 and CYG-2 were each recovered from a single goat specimen. Genotype CHG1 was detected in all three goat-sampling regions, while the remaining caprine genotypes exhibited restricted geographical circulation (Table 1).

### 3.3. Phylogenetic Classification

**Figure 2:**
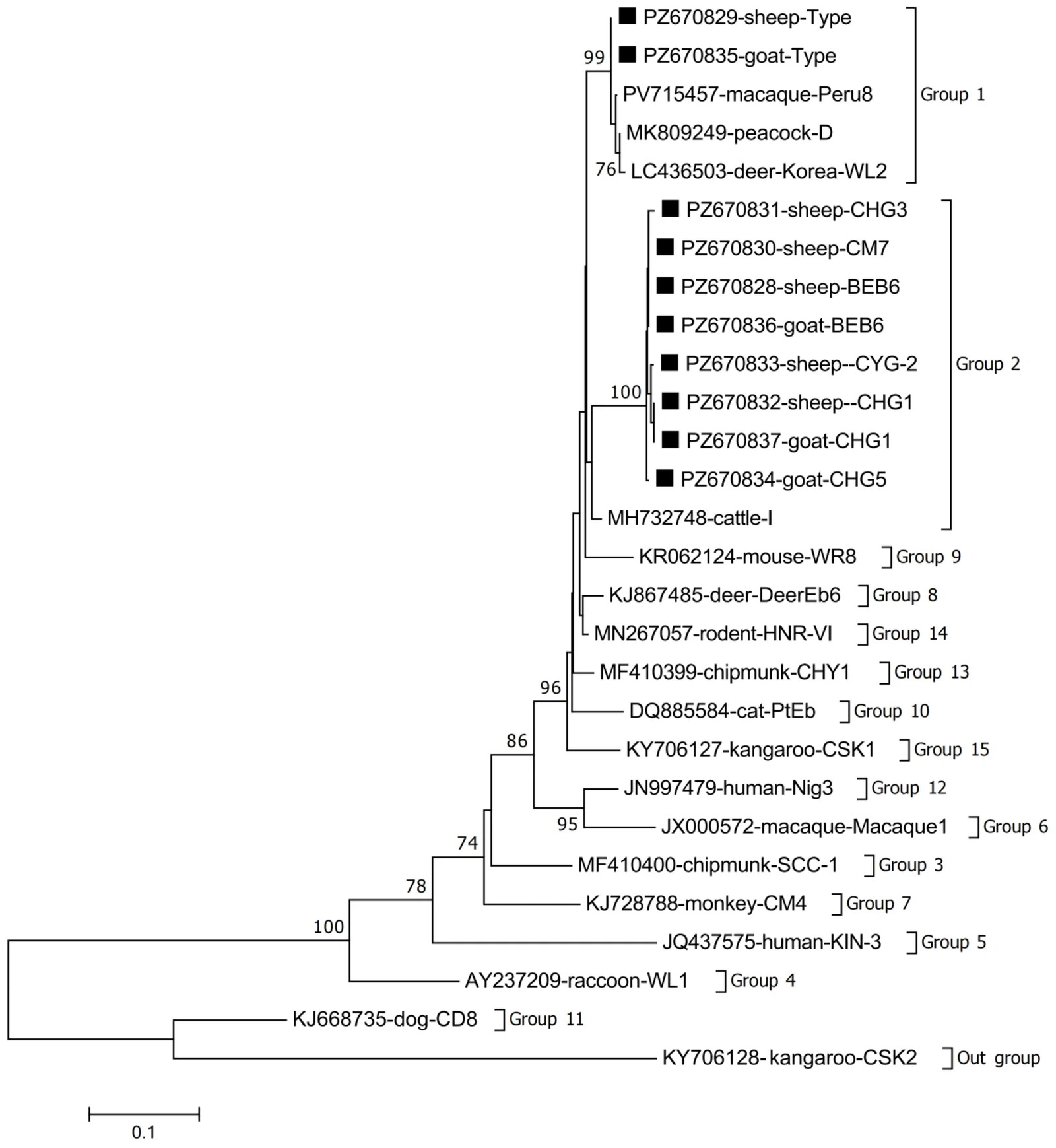
Neighbor-joining (NJ) phylogenetic tree constructed based on the ITS nucleotide sequences of *E. bieneusi*. Bootstrap values (>70%) calculated from 1000 replicates are shown at each node to assess tree reliability. All genotypes identified in the present study are marked with black squares ⬛. Reference isolates are labeled with their GenBank accession numbers, host species, and genotype names. Genotypes were categorized into 15 distinct phylogenetic groups (Group 1–Group 15); the zoonotic Group 1 and Group 2 clades containing our obtained sequences are shaded in different colors. *Nosema bombycis* was used as the outgroup for tree rooting.

Phylogenetic reconstruction based on aligned ITS sequences revealed that all seven recovered genotypes clustered within two major zoonotic clades. Genotype Type IV fell into Group 1, the canonical clade harboring the majority of globally documented human-pathogenic isolates. The remaining six genotypes (CM7, BEB6, CHG1, CHG3, CHG5, CYG-2) grouped within Group 2, a ruminant-associated clade with well-documented emerging zoonotic capacity (Figure 2).

## 4. Discussion

To the best of our knowledge, this study provides the first molecular epidemiological data on *E. bieneusi* in Hu sheep, and the first parallel comparative analysis of sympatric sheep and goat populations in eastern China. The findings reveal notable inter-species differences in infection prevalence, age susceptibility and dominant genotype profiles, which may be related to the distinct intensive indoor and semi-free-range rearing practices used for the two breeds. The overall *E. bieneusi* prevalence of 24.53% in Hu sheep falls within the medium-to-high range of global ovine estimates, exceeding the global pooled prevalence of 17.4% reported in a recent systematic meta-analysis [9]. Globally, the Hu sheep prevalence in this study was higher than those reported in sheep from Portugal (2.0%), Egypt (6.7%), Türkiye (8.0%), Iran (12.0%), and Brazil (19.2%) [14,15,16,17,18]. Nevertheless, this rate was substantially lower than the extremely high prevalence of 68.0% in Swedish sheep [19]. For goats, the present regional prevalence of 10.87% was lower than those documented in Thai goats (19.2%) [20] and Iranian goats (26.1%) [17], while being higher than the low baseline prevalence of 6.4% reported in Portuguese goat populations [21].

Nationwide comparative analysis revealed pronounced geographical heterogeneity of *E. bieneusi* prevalence across China, with distinct disparities among different geographical regions and provinces. The overall prevalence of *E*. *bieneusi* among Hu sheep in southern Zhejiang reached 24.5%, a value comparable to the 23.4% infection rate reported in Tibetan sheep on the Qinghai–Tibet Plateau [22]. This prevalence was markedly higher than those documented in local sheep herds from Liaoning (9.4%) [23]. Nevertheless, our detection rate was substantially lower than the high infection levels recorded in pastoral northern China, including Ningxia (41.1%) [24], Inner Mongolia (76.7%) [25] and Gansu (34.5%) [26]. For goats, the 10.87% prevalence obtained herein was higher than the rates reported in Anhui goats (4.6%) [23] and Tibetan goats (9.6%) [27], and considerably lower than the high-prevalence provinces such as Hainan (25.7%) [28], Jiangsu (35.5%) [29] and Henan (32.2%) [23]. Notably, a previous molecular epidemiological survey conducted across northern and central Zhejiang reported a caprine *E. bieneusi* prevalence of 19.2%, nearly twice the value detected in southern Zhejiang in the current investigation, which confirms obvious spatial variation in pathogen endemicity within Zhejiang Province of China [10].

The geographical disparities in *E. bieneusi* prevalence may be partially associated with divergent breeding modes, farm management, and microenvironmental conditions, alongside differences in stocking density, facility infrastructure and sampling timing across individual farms. Notably, higher-prevalence regions (Cangnan, Taishun) had higher stocking densities and older facilities compared to lower-prevalence regions. Additionally, although sampling spanned multiple seasons for all regions, exact timing varied by farm and may introduce minor confounding. Northern and western Chinese pastoral areas (e.g., Inner Mongolia, Gansu, Ningxia) predominantly adopt extensive free-grazing systems, which may increase livestock exposure to environmental spore contamination, wild animal carriers and mixed flocks, potentially contributing to higher infection risk [24,25,26]. In contrast, livestock breeding in Zhejiang Province relies on standardized intensive indoor feeding, with regular environmental disinfection and unified manure disposal, which may reduce external pathogen introduction and environmental transmission pressure, potentially contributing to the generally lower prevalence observed in eastern China [23]. Nonetheless, the high indoor stocking density of Hu sheep breeding barns may facilitate rapid horizontal intra-herd transmission if sporadic infection occurs, which could help maintain a moderate endemic prevalence and prevents the regional infection rate from dropping to an extremely low level.

Age exhibited a prominent association with *E. bieneusi* prevalence in Hu sheep, with pre-weaning lambs presenting a markedly higher infection rate (60.42%) than older individuals. Given the cross-sectional design, we cannot disentangle true age effects from birth cohort effects or age-specific management practices. In the enrolled intensive Hu sheep farms, lambs, fattening sheep and adult ewes are routinely housed in separate barns with distinct feeding and cleaning protocols, which may act as a confounding factor for the observed age-related prevalence gradient. This age-related epidemiological pattern is consistent with previous surveys concerning small ruminant microsporidiosis in China [23,29]. The elevated infection susceptibility of young lambs is biologically plausible, which may be partly related to their immature immune function, potentially increasing vulnerability to opportunistic pathogen infection. Moreover, intensive indoor rearing and frequent oral exploratory behaviors in young lambs may facilitate fecal–oral transmission, potentially accelerating *E. bieneusi* spread within flocks [9]. Additionally, co-infection with other common enteric pathogens of young lambs, such as *Cryptosporidium* spp. and *Giardia duodenalis*, may impair intestinal epithelial barrier function and further increase susceptibility to *E. bieneusi* infection; this hypothesis warrants investigation in future multi-pathogen cohort studies. Most previous Chinese studies have documented the highest *E. bieneusi* prevalence in post-weaning lambs aged 3–6 months, a pattern that has been attributed to the decline of maternal antibody protection after weaning [23,24,29]. Notably, this study identified an infection peak in pre-weaning lambs aged < 3 months. This discrepancy may be related to the ultra-high stocking density of local intensive Hu sheep farms, which could enable efficient horizontal pathogen transmission and induces early-stage endemic infection in pre-weaning lambs before maternal immunity wanes. In contrast, no significant age-associated correlation was observed for *E. bieneusi* infection in goats. The lack of statistical significance may be partly due to the moderate sample size and simplified binary age grouping, which may limit the statistical power to detect subtle age-based differences. Furthermore, divergent rearing patterns may partly explain the distinct age-susceptibility profiles between the two host species. Unlike fully confined Hu sheep lambs, juvenile goats are mainly raised under semi-free-range conditions with lower population density and wider activity ranges, which may reduce horizontal transmission pressure and lowers early-life infection risk [30,31]. Collectively, these correlational findings highlight age-dependent infection characteristics in Hu sheep, while further longitudinal and multivariate analyses are required to verify the independent effects of age on *E. bieneusi* susceptibility.

At the genotypic level, seven distinct known *E. bieneusi* ITS genotypes were identified across the two host species, with obvious host-specific dominance patterns. CM7 and BEB6 predominated in Hu sheep, while CHG1 was the dominant genotype in goats. Three zoonotic genotypes (BEB6, Type IV and CHG1) were detected in both Hu sheep and goats, suggesting possible shared environmental exposure or inter-species circulation between sympatric small ruminant populations. Shared genotypes alone cannot confirm direct cross-species transmission, as independent pathogen introduction and regional endemic circulation of widespread genotypes represent alternative explanations. Considering the frequent live animal transfer and mixed grazing activities across adjacent farms in southern Zhejiang, shared genotypes likely reflect ongoing inter-herd and inter-species pathogen flow, rather than independent introduction events. All seven detected genotypes were clustered within Group 1 and Group 2, the two major groups universally recognized for zoonotic *E. bieneusi* lineages, indicating that the locally circulating isolates possess considerable zoonotic potential. Notably, ITS-based genotyping has limited discriminatory power for fine-scale transmission tracking and population structure analysis, and cannot definitively verify direct transmission events.

Type IV, a typical Group 1 genotype, exhibits an extremely broad host range and has been widely detected in human diarrheal patients and over 60 animal species worldwide, including mammals, birds and wild animals [2]. It is frequently involved in waterborne and food-borne microsporidiosis outbreaks across diverse geographical regions [3,6,7]. Although Type IV was detected only sporadically in a single Hu sheep and a single goat in the present study, its identification is of notable public health importance. This finding confirms the circulation of highly adaptive, pan-zoonotic *E. bieneusi* strains in local livestock. Infected animals continuously shed viable spores into the surrounding environment, which may facilitate environmental contamination and create potential transmission routes from livestock to local human residents via polluted water, soil, or agricultural products. BEB6 is one of the most prevalent and geographically widespread *E. bieneusi* genotypes in global small-ruminant populations [2,9]. Although historically regarded as ruminant-specific, BEB6 has been repeatedly identified in human diarrheal cases, firmly confirming its zoonotic capacity [2,32]. The co-circulation of BEB6 in both Hu sheep and goats in this region constitutes a persistent exposure risk for occupational groups, including farmers, breeders, and veterinary staff, who have frequent direct contact with livestock and contaminated environments [32].

CM7 was the overwhelmingly dominant genotype in Hu sheep, accounting for nearly two-thirds of all ovine isolates, and was widely distributed across all surveyed regions and age groups. This genotype was initially identified in non-human primates and was simultaneously reported as COS-1 in sheep from northeastern China [30,33]. Subsequent investigations have confirmed its circulation across multiple ungulate species in northern and western China, but it had never been documented in eastern coastal regions prior to this work. Our finding therefore not only expands the known host range of CM7 to include Hu sheep, but also fills a geographical gap in its distribution across China, suggesting that this genotype may have been introduced to eastern China via live animal trade, though this hypothesis remains speculative without formal herd traceability data. Given its high prevalence and wide distribution across all age groups and sampling regions, CM7 should be prioritized as a core endemic genotype for routine molecular surveillance in intensive Hu sheep production systems.

Genotypes CHG1, CHG3, and CHG5 were initially identified in Chinese goats [23]. Subsequent epidemiological investigations have detected these genotypes in a wide range of herbivorous animals, including cattle, sheep, and deer, as well as in non-human primates, dogs, and poultry such as geese [2,34]. Notably, CHG3 and CHG5 have been repeatedly detected in human populations, indicating considerable zoonotic potential [35,36]. In Hainan Province, CHG3 was isolated from five diarrheal patients, while CHG5 was identified in six diarrheal and four asymptomatic individuals [35]. Moreover, CHG3 has been recognized as a predominant genotype in diarrheal patients in Shanghai, and CHG5 has also been previously detected in local diarrheal populations in the present study region [36]. Collectively, these findings are consistent with the hypothesis of potential animal-to-human transmission of CHG3 and CHG5, particularly spillover between small ruminants and human populations. In contrast, CYG-2 was first characterized in Yunnan black goats and exhibits obvious host tropism for caprine animals [37]. To date, CYG-2 has not been detected in humans or non-caprine hosts, suggesting a currently low zoonotic risk. However, the limited host range of CYG-2 may merely reflect insufficient sampling and epidemiological surveillance. With expanding molecular epidemiological investigations, further host diversity of this genotype is likely to be uncovered. Considering that host-range expansion is a common evolutionary feature of *E. bieneusi*, continuous monitoring of this rare caprine-specific genotype is still necessary.

Occupational populations, including farm workers, veterinarians and slaughterhouse personnel, face particularly high exposure risk due to frequent direct contact with infected animals and feces. From a One Health perspective, the continuous circulation of these confirmed zoonotic genotypes poses dual public health risks. On one hand, occupational populations including farm workers, veterinarians and slaughterhouse personnel face direct exposure risks via close contact with infected animals and feces. On the other hand, spore shedding from livestock may contaminate surrounding water sources and soil, creating environmental transmission pathways for nearby residential populations.

These epidemiological and genotypic findings have practical implications for on-farm biosecurity management. For intensively housed Hu sheep, targeted interventions should focus on pre-weaning lamb populations: implement routine targeted molecular screening for pre-weaning lamb cohorts, house different age groups in physically separate units, enforce daily cleaning and scheduled disinfection of lamb pens, and adopt standardized composting or anaerobic treatment of manure before agricultural use to reduce environmental spore shedding. For semi-free-range goats, priority measures include: regular pathogen testing of drinking water sources, physical separation of grazing areas from other ruminant herds to avoid mixed grazing, and mandatory quarantine and *E. bieneusi* screening for all newly introduced animals before entering the flock. For public health protection, we recommend: targeted occupational health education for farm workers and veterinary staff to raise awareness of fecal–oral transmission risks; periodic voluntary fecal screening for high-risk occupational populations; and integrated surveillance programs that combine livestock pathogen monitoring, environmental water and soil testing, and human clinical case tracking to comprehensively map transmission chains at the human–animal-environment interface. Routine molecular monitoring of *E. bieneusi* in both livestock and environmental matrices is recommended to dynamically evaluate infection status and adjust prevention strategies accordingly.

### 4.1. Study Limitations

This study has several important limitations that should be acknowledged. First, this was a cross-sectional observational study, and all identified associations between prevalence and host demographics, geography, or rearing systems are correlational and cannot be interpreted as causal relationships. We were unable to disentangle age effects from cohort or management effects in Hu sheep, and no multivariable regression analysis was performed to identify independent risk factors. Second, systematic quantitative data on farm-level management practices (including stocking density, hygiene protocols, water sources, and manure management) were not collected, limiting our ability to directly evaluate the impact of rearing systems on infection risk. Third, no environmental samples (water, soil, or manure) were analyzed, so the role of environmental contamination in pathogen transmission could not be directly assessed. Fourth, genotyping was based exclusively on the ITS region, which has limited discriminatory power for fine-scale transmission tracking and population structure analysis; shared genotypes therefore cannot confirm direct cross-species transmission. Fifth, age stratification was inconsistent between the two host species due to incomplete breeding records for goats, reducing the comparability of age-related infection patterns between sheep and goats. Sixth, goat sampling was not conducted in Cangnan County due to logistical constraints, which reduces full geographical parallel comparability between the two host species across all four regions. Finally, the small number of farms per region means we cannot rule out farm-level cluster effects, and observed regional differences may partially reflect individual farm characteristics rather than purely geographic variation.

### 4.2. Future Research Directions

Building on the baseline data provided by this study, further research is warranted to deepen the understanding of pathogen transmission dynamics and associated public health risks in this region. First, longitudinal cohort studies combined with multivariate regression models will be conducted to verify the causal relationships between potential risk factors (e.g., age, rearing pattern, regional environment) and *E. bieneusi* infection, and to statistically validate the independent effects of demographic and geographical variables on infection susceptibility. Second, subsequent surveillance will expand sampling coverage to more prefectures across Zhejiang Province and adjacent eastern Chinese regions with a more representative farm sampling framework, to improve the generalizability of epidemiological findings and clarify the spatial transmission patterns of *E. bieneusi* at a larger geographical scale. Third, whole-genome sequencing-based analyses will be incorporated in future investigations to further explore the genetic diversity, evolutionary characteristics and fine-scale transmission trajectories of local *E. bieneusi* isolates, beyond the genotypic resolution provided by the single ITS marker. Finally, integrative surveillance covering clinical symptom tracking in infected animals, spore detection in environmental matrices (water, soil and manure), and human population screening in occupational exposure groups will be established to comprehensively decipher the complete transmission chains of *E. bieneusi* at the human–livestock–environment interface of this region.

## 5. Conclusions

This molecular surveillance confirmed endemic circulation of *E. bieneusi* among Hu sheep and goat populations in southern Zhejiang, with a significantly higher infection prevalence observed in sheep than in sympatric goats. Epidemiological correlates indicated that geographical distribution and host age exhibited observational associations with *E. bieneusi* infection levels in Hu sheep, whereas no obvious age-related disparity was detected in goats. Genotypic analysis identified seven distinct ITS genotypes, all of which were phylogenetically affiliated with the zoonotic Group 1 and Group 2 lineages. Host-specific genotype predominance was clearly observed: CM7 and BEB6 were the dominant genotypes in Hu sheep, while CHG1 prevailed in local goat populations. The persistent circulation of verified zoonotic genotypes, including Type IV, BEB6, CHG3, and CHG5, suggests that farmed small ruminants serve as important reservoir hosts for human microsporidiosis in this region, posing potential occupational and environmental public health risks. Collectively, these findings fill the epidemiological gap of *E. bieneusi* in Hu sheep and establish a comparative baseline for sympatric small ruminants in eastern China. The identified host-specific infection patterns and potentially rearing-related epidemiological characteristics provide a scientific basis for formulating breed-tailored biosecurity measures. The confirmed circulation of multiple zoonotic genotypes and potential cross-species transmission risk highlight the necessity of integrating livestock surveillance, environmental management and human occupational protection into local microsporidiosis control, under a unified One Health strategy.

## Figures and Tables

**Table 1:** Prevalence and genotype distribution of *E. bieneusi* in Hu sheep and goats in southern Zhejiang, China.

Category	Hu Sheep			Goats		
	No. Examined	No. Positive (% [95% CI])	Genotypes (n)	No. Examined	No. Positive (% [95% CI])	Genotypes (n)
Regional distribution						
Taishun	32	13 (40.63 [25.5–57.8])	BEB6 (3), CM7 (10)	43	4 (9.30 [3.7–21.9])	Type IV (1), BEB6 (3)
Cangnan	46	20 (43.48 [29.8–58.0])	BEB6 (6), CM7 (14)	—	—	—
Yueqing	43	4 (9.30 [3.7–21.9])	CHG1 (2), BEB6 (1), CM7 (1)	40	3 (7.50 [2.6–20.0])	CHG3 (1), CYG-2 (1), CHG1 (1)
Taizhou	38	2 (5.26 [1.5–17.3])	CHG5 (1), Type IV (1)	55	8 (14.55 [7.3–27.0])	CHG1 (8)
Age distribution						
<3 months	48	29 (60.42 [45.6–73.6])	BEB6 (9), CM7 (20)	—	—	—
4 months–1 year	44	7 (15.91 [7.9–29.6])	CM7 (4), CHG1 (2), BEB6 (1)	62	7 (11.29 [5.4–22.2])	Type IV (1), BEB6 (2), CHG3 (1), CYG-2 (1), CHG1 (2)
>1 year	67	3 (4.48 [1.4–12.5])	CM7 (1), Type IV (1), CHG5 (1)	76	8 (10.53 [5.2–20.1])	BEB6 (1), CHG1 (7)
Total	159	39 (24.53 [18.2–32.0])	CM7 (25), BEB6 (10), CHG1 (2), CHG5 (1), Type IV (1)	138	15 (10.87 [6.6–17.3])	CHG1 (9), BEB6 (3), CHG3 (1), CYG-2 (1), Type IV (1)

Note: No., number; n, number of isolates; —, no sample collected in this category; CI, confidence interval.

## Data Availability

All ITS nucleotide sequences generated in this study have been deposited in the GenBank public repository under accession numbers PZ670828–PZ670837, covering 7 distinct genotypes with separate submissions for host-specific isolates of the three shared genotypes. All raw analytical datasets supporting the conclusions presented within this manuscript are fully included in the article text.
